# Electronic health record tools to assist with children’s insurance coverage: a mixed methods study

**DOI:** 10.1186/s12913-018-3159-x

**Published:** 2018-05-10

**Authors:** Jennifer E. DeVoe, Megan Hoopes, Christine A. Nelson, Deborah J. Cohen, Aleksandra Sumic, Jennifer Hall, Heather Angier, Miguel Marino, Jean P. O’Malley, Rachel Gold

**Affiliations:** 1 OCHIN, Inc., 1881 SW Naito Parkway, Portland, OR 97201 USA; 2 Department of Family Medicine, Oregon Health & Science University, 3181 Sam Jackson Road, Mail Code FM, Portland, OR 97239 USA; 3 Kaiser Permanente Northwest Center for Health Research, 3800 N Interstate Avenue, Portland, OR 97211 USA

**Keywords:** Children, Health insurance, Medicaid, CHIP, Community health centers, Electronic health record

## Abstract

**Background:**

Children with health insurance have increased access to healthcare and receive higher quality care. However, despite recent initiatives expanding children’s coverage, many remain uninsured. New technologies present opportunities for helping clinics provide enrollment support for patients. We developed and tested electronic health record (EHR)-based tools to help clinics provide children’s insurance assistance.

**Methods:**

We used mixed methods to understand tool adoption, and to assess impact of tool use on insurance coverage, healthcare utilization, and receipt of recommended care. We conducted intent-to-treat (ITT) analyses comparing pediatric patients in 4 intervention clinics (*n* = 15,024) to those at 4 matched control clinics (*n* = 12,227). We conducted effect-of-treatment-on-the-treated (ETOT) analyses comparing intervention clinic patients with tool use (*n* = 2240) to intervention clinic patients without tool use (n = 12,784).

**Results:**

Tools were used for only 15% of eligible patients. Qualitative data indicated that tool adoption was limited by: (1) concurrent initiatives that duplicated the work associated with the tools, and (2) inability to obtain accurate insurance coverage data and end dates. The ITT analyses showed that intervention clinic patients had higher odds of gaining insurance coverage (adjusted odds ratio [aOR] = 1.32, 95% confidence interval [95%CI] 1.14–1.51) and lower odds of losing coverage (aOR = 0.77, 95%CI 0.68–0.88), compared to control clinic patients. Similarly, ETOT findings showed that intervention clinic patients with tool use had higher odds of gaining insurance (aOR = 1.83, 95%CI 1.64–2.04) and lower odds of losing coverage (aOR = 0.70, 95%CI 0.53–0.91), compared to patients without tool use. The ETOT analyses also showed higher rates of receipt of return visits, well-child visits, and several immunizations among patients for whom the tools were used.

**Conclusions:**

This pragmatic trial, the first to evaluate EHR-based insurance assistance tools, suggests that it is feasible to create and implement tools that help clinics provide insurance enrollment support to pediatric patients. While ITT findings were limited by low rates of tool use, ITT and ETOT findings suggest tool use was associated with better odds of gaining and keeping coverage. Further, ETOT findings suggest that use of such tools may positively impact healthcare utilization and quality of pediatric care.

**Trial registration:**

ClinicalTrials.gov, NCT02298361; retrospectively registered on November 5, 2014.

**Electronic supplementary material:**

The online version of this article (10.1186/s12913-018-3159-x) contains supplementary material, which is available to authorized users.

## Background

Health insurance coverage is associated with increased access to healthcare and reduced unmet needs [1–4]. Insured children are more likely to receive recommended preventive care and less likely to experience preventable hospitalizations compared to uninsured children [5–10]. In the United States (US), although children’s coverage steadily increased following Children’s Health Insurance Program (CHIP) expansions, [11] important gaps remain [12–15]. For example, 28% of children insured by Medicaid or CHIP lost coverage within 12 months, [14] and two years after passage of the CHIP Reauthorization Act, 21% of pediatric patients seen in a community health center (CHC) network were uninsured [16].

Concurrent to CHIP expansions, technological developments have yielded new opportunities to enhance how healthcare teams support patients with gaining insurance coverage [17–20]. To act on these developments, we conducted the Innovative Methods for Parents And Clinics to Create Tools for Kids’ Care (IMPACCT Kids’ Care) Study, [21] in which we built electronic health record (EHR) tools to help primary care CHCs provide health insurance enrollment assistance. We chose this setting because many low-income children eligible for Medicaid or CHIP receive care at CHCs [22]. The tools were designed to help CHC staff monitor patients’ coverage eligibility and track provided insurance support. To our knowledge, no previous efforts have sought to develop, implement, and study such tools in CHCs or any other settings. Thus this research, while exploratory, is highly novel.

## Methods

### Overview

We implemented the EHR-based tools in four intervention CHCs and selected four matched control CHCs for comparison. We used a retrospective cohort design to identify our pediatric study population (aged 0–19 at time of visit). We measured tool use; assessed facilitators and barriers to tool use; and assessed tool impact and effect on health insurance coverage, CHC utilization, and receipt of recommended care. We used intent-to-treat (ITT) analyses to measure tool impact *at the population level*. We used effect-of-treatment-on-the-treated (ETOT) analyses to measure what happened *when the tools were used* and to distinguish consideration of the tools’ effect on individuals from consideration of the tools’ effect on populations. Details of the study design and tool development processes were previously described [21, 23, 24].

### Study period

The tools were implemented on 6/1/2014; we collected and analyzed data from 6 months pre-implementation through 18 months post-implementation.

### Setting

Participating CHCs were members of OCHIN, Inc., a 501(c)(3) organization that provides health information technology to CHCs [25, 26]. The majority of these CHCs’ pediatric patients met income eligibility for Medicaid or CHIP, yet many were uninsured at the time of a visit, and thus could benefit from the tools. OCHIN members share an Epic© EHR, making it possible to build and implement the study tools on a single EHR platform, and centrally obtain EHR data.

### Sample

Eight CHCs participated in this study: four Oregon CHCs volunteered to be ‘intervention’ sites, and four ‘control’ sites were selected from a pool of 38 non-intervention CHCs in Oregon using a propensity score technique [27] to match sites by patient population demographics (ratio of children to adults and percent Hispanic ethnicity) and date of EHR implementation (number of months of clinic EHR experience). The clinic with the closest propensity score was selected as the matched control for each intervention clinic. In addition to matching, we controlled for differences between intervention and control sites (i.e., residual confounding) through statistical regression adjustment.

### Intervention

We developed the EHR tools in OCHIN Epic© via a user-centered design process, described elsewhere [23, 24]. Detailed tool descriptions are provided in Additional file 1. The central tool was a health insurance assistance tracking form in a patient’s medical record; we used data from this tool to determine that patients had been exposed to the intervention. In addition to the tracking form, pop-up alerts were built into the EHR to notify staff when a patient’s insurance was soon to expire or when a patient appeared eligible but was not enrolled in public insurance. These were visible in the EHR at all relevant patient visits. A data roster tool was also available for CHC staff to create lists of patients for whom insurance assistance had been initiated and required follow-up, or who had an upcoming appointment and might need enrollment support. The tools were designed with low-income pediatric patients in mind but were available for use on any patients at the intervention clinics.

### EHR and Medicaid datasets

We used patient-level EHR data to assess whether or not the health insurance assistance tracking form was used, and to obtain demographic and visit data. We also used EHR data to assess coverage status at visits. To quantify longitudinal insurance coverage periods (coverage start and end dates), we created individual patient linkages between EHR data and Medicaid/CHIP enrollment data from the state of Oregon, per established methods using Medicaid/CHIP identification numbers [28–30]. We used household case numbers in the Medicaid/CHIP enrollment data to measure how long each household was established at a clinic.

### Variables

The primary predictor variable for ITT analyses was whether a pediatric patient received care at one of the four intervention clinics versus one of the four control clinics. The primary predictor variable for ETOT analyses was whether or not a pediatric patient in the intervention clinics had the tracking tool used to assist them or not. Other covariates are shown in the tables. Outcome variables were: health insurance coverage status, coverage gain, coverage loss, utilization of care, and receipt of recommended care (assessed using healthcare quality measures from the Children’s Health Insurance Program Reauthorization Act (CHIPRA)) [31].

### Analyses

**Fig. 1:**
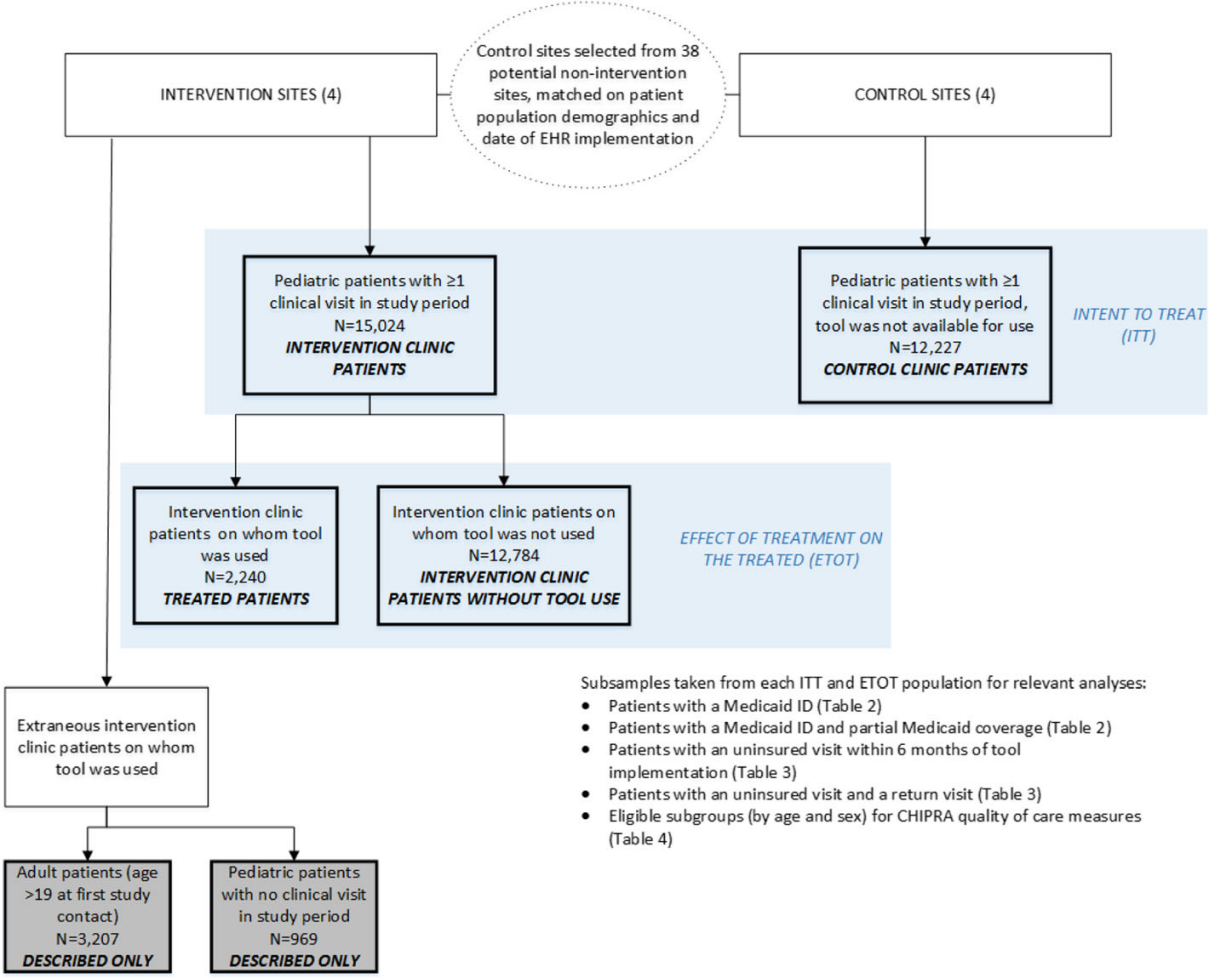
Overview of study groups. Description of comparison groups and processes utilized for quantitative assessment

We used mixed methods to measure rates of tool use and to understand facilitators and barriers to tool use [32]. We used ITT and ETOT analyses to assess the impact of the tools on health insurance coverage, CHC utilization, and receipt of recommended healthcare services. In ITT analyses, we compared pediatric patients with ≥1 clinical visit at an intervention clinic (*n* = 15,024) to those with ≥1 clinical visit at a matched control CHC (*n* = 12,227); see Fig. 1. In ETOT analyses, among all pediatric patients with ≥1 clinical visit at an intervention clinic, we compared those patients on whom the tracking tool was used (*n* = 2240) with those on whom the tool was not used (ETOT comparisons, n = 12,784); Fig. 1. To assess outcomes related to coverage and utilization, we conducted several of the analyses in subgroups of patients for whom we had Medicaid data (described below).

#### Mixed methods to assess tool use

##### Quantitative

We calculated rate of tool use as the number of pediatric patients with ≥1 clinical visit at an intervention CHC for whom the tracking tool was used (numerator = 2240) over the total number of intervention clinic pediatric patients with ≥1 clinical visit at an intervention CHC (denominator = 15,024). Using chi-square tests, we compared demographic, care utilization, and coverage characteristics of (1) intervention clinic patients versus control clinic patients (ITT groups), and (2) patients at the intervention clinics with tool use versus patients at the intervention clinics without tool use (ETOT groups). We adjusted for statistically significant differences between groups using multivariable analyses (described below).

##### Qualitative

Prior to tool implementation, we conducted observations and interviews at two intervention and two comparison CHCs, purposively selected to assess baseline insurance assistance processes. Semi-structured interviews were conducted with clinic leaders, clinicians, and staff (*N* = 26, 5–7 individuals per site) to understand the clinics’ insurance assistance processes and inform tool development [23]. One year after tool implementation, we conducted observations and interviews at the intervention sites to observe insurance assistance processes and tool use. We spent 2–3 days at each clinic, observing processes and creating detailed fieldnotes. Field researchers also conducted semi-structured interviews with intended tool users (e.g., front desk staff, schedulers, eligibility specialists / insurance assistants, clinic administrators; *N* = 12) to assess perceptions of the tools. These interviewees were purposively selected among staff observed to have tasks most relevant to assisting patients with health insurance, regardless of whether these staff members were using the tools or not using them.

Recorded interviews were professionally transcribed. Transcripts and fieldnotes were de-identified and entered into Atlas.ti (Version 7.0, Atlas.ti Scientific Software Development GmbH, Berlin, Germany) for analysis. We used a grounded theory approach to analyze the data [33]. First, our multi-disciplinary research team met regularly to analyze all data for one clinic, informing how we named and tagged the text, and how we defined emerging themes to create a code book. We repeated this process for each clinic, meeting to analyze and make sense of the data. Then, we compared emerging themes across the clinics, and identified factors that influenced clinic tool use.

#### ITT and ETOT analyses: Tool impact on coverage, utilization, receipt of recommended care

For both ITT and ETOT analyses, our assessment period included the 6 months prior to tool implementation, which allowed us to incorporate patients who would have been identified as uninsured or about to lose coverage, were the tool used proactively. To examine changes in *coverage status* and *clinic utilization*, we calculated odds ratios comparing ITT and ETOT groups using adjusted logistic generalized estimating equation (GEE) models to account for clustering of patients within clinic, and adjusted for covariates (see footnotes, Tables 2 and 3). Where there were statistically significant demographic and utilization differences between patients in comparison groups (Table 1), we adjusted for these factors in the analyses. Coverage and utilization outcomes were conducted in relevant subsets of patients as follows:

1. Among patients who could be linked to Medicaid/CHIP data (> 90% of patients), we assessed (a) the odds of gaining coverage after a period of uninsurance, and (b) the odds of losing coverage after having Medicaid/CHIP coverage at any time during the study period. Medicaid/CHIP enrollment data needed to evaluate this outcome were available through 9/30/2015, so these analyses ended at that date (Table 2).
2. Among patients with an uninsured visit within 6 months of the tool’s implementation, we calculated the odds of returning for a second visit. This analysis utilized visit coverage information from the EHR and was not limited to patients who could be linked to Medicaid/CHIP (Table 3).
3. Among patients in (2) who did return for a visit, we calculated the (a) odds of being uninsured and (b) the odds of being insured by Medicaid/CHIP at return visit(s) (Table 3).

**Table 1:** Demographic and encounter characteristics among ITT and ETOT study groups

	Intent To Treat (ITT)			Effect of Treatment on the Treated (ETOT) Intervention clinic patients only		
	Intervention clinic patients^a^ *N* = 15024	Control clinic patients^b^ *N* = 12227	*p*-value^c^	Intervention clinic patients with tool use^d^ *N* = 2240	Intervention clinic patients without tool use^e^ N = 12784	*p*-value^c^
Sex			0.22			0.347
Male	7381 (49.1)	6099 (49.9)		1121 (50.0)	6260 (49.0)	
Female	7643 (50.9)	6128 (50.1)		1119 (50.0)	6524 (51.0)	
Age (at first visit or tool usage)			<.001			<.001
0–12 mo.	1471 (9.8)	1452 (11.9)		74 (3.3)	1397 (10.9)	
1–4 yrs.	2902 (19.3)	2595 (21.2)		449 (20.0)	2453 (19.2)	
5–12 yrs.	6131 (40.8)	4807 (39.3)		994 (44.4)	5137 (40.2)	
13–17 yrs.	3387 (22.5)	2483 (20.3)		558 (24.9)	2829 (22.1)	
18–19 yrs.	1133 (7.5)	890 (7.3)		165 (7.4)	968 (7.6)	
Race-ethnicity			<.001			<.001
Hispanic	10398 (69.2)	7266 (59.4)		2128 (95.0)	8270 (64.7)	
Non-Hispanic white	3501 (23.3)	2811 (23.0)		73 (3.3)	3428 (26.8)	
Non-Hispanic other	680 (4.5)	1968 (16.1)		29 (1.3)	651 (5.1)	
Unknown	445 (3.0)	182 (1.5)		10 (0.5)	435 (3.4)	
Primary language			<.001			<.001
Spanish	8466 (56.4)	5803 (47.5)		1976 (88.2)	6,90 (50.8)	
English	5870 (39.1)	5103 (41.7)		241 (10.8)	5629 (44.0)	
Other	644 (4.3)	1125 (9.2)		22 (1.0)	622 (4.9)	
Unknown	44 (0.3)	196 (1.6)		1 (0.04)	43 (0.3)	
Percent of federal poverty level			<.001			<.001
≤138	13336 (88.8)	9998 (81.8)		2083 (93.0)	11253 (88.0)	
139–199	848 (5.6)	918 (7.5)		114 (5.1)	734 (5.7)	
≥200	312 (2.1)	1204 (9.9)		29 (1.3)	283 (2.2)	
Unknown	528 (3.5)	107 (0.9)		14 (0.6)	514 (4.0)	
N encounters in study period^f^			0.09			<.001
1	5408 (36.0)	4525 (37.0)		652 (29.1)	4756 (37.2)	
2	3536 (23.5)	2756 (22.5)		556 (24.8)	2980 (23.3)	
≥3	6080 (40.5)	4946 (40.5)		1032 (46.1)	5048 (39.5)	
New/established patient at first encounter			<.001			<.001
New patient	3510 (23.4)	2486 (20.3)		258 (11.5)	3252 (25.4)	
Established patient	11514 (76.6)	9741 (79.7)		1982 (88.5)	9532 (74.6)	
Have a Medicaid identification number (ID)			<.001			<.001
Yes	13614 (90.6)	11729 (95.9)		2080 (92.9)	11534 (90.2)	
No	1410 (9.4)	498 (4.1)		160 (7.1)	1250 (9.8)	

Data source: EHR

^a^All intervention clinic patients with ≥1 clinical encounter in study period

^b^All control clinic patients with ≥1 clinical encounter in study period

^c^χ^2^ tests of independence

^d^Intervention clinic patients with ≥1 clinical encounter in study period with tool use

^e^Intervention clinic patients with ≥1 clinical encounter in study period without tool use

^f^Tool implementation date (6/1/2014) through 18 months post-implementation (11/30/2015)

**Table 2:** Medicaid measures and coverage status, in the subset of patients with a Medicaid ID

	Intent To Treat (ITT)			Effect of Treatment on the Treated (ETOT) Intervention clinic patients only		
	Intervention clinic patients with Medicaid ID *N* = 13614	Control clinic patients with Medicaid ID *N* = 11729	*p*-value^a^	Patients with Medicaid ID with tool use *N* = 2080	Patients with Medicaid ID without tool use N = 11534	*p*-value^a^
Medicaid case characteristics and coverage status						
Other family member(s) on case, N (%)			<.001			<.001
Yes	11547 (84.8)	10288 (87.7)		1848 (88.9)	9699 (84.1)	
No	2067 (15.2)	1441 (12.3)		232 (11.1)	1835 (15.9)	
How long the family was established with clinic, at study start date, N (%)			<.001			<.001
Not established	2164 (15.9)	1856 (15.8)		113 (5.4)	2051 (17.8)	
< 1 yr.	697 (5.1)	645 (5.5)		41 (2.0)	656 (5.7)	
1 - < 3 yrs.	1402 (10.3)	1496 (12.8)		149 (7.2)	1253 (10.9)	
3 - < 6 yrs.	2162 (15.9)	1989 (17.0)		293 (14.1)	1869 (16.2)	
≥6 yrs.	7189 (52.8)	5743 (49.0)		1484 (71.4)	5705 (49.5)	
Percent of study period^b^ covered by Medicaid, N (%)			<.001			<.001
0%	481 (3.5)	297 (2.5)		30 (1.4)	451 (3.9)	
< 50%	1175 (8.6)	933 (8.0)		113 (5.4)	1062 (9.2)	
50–99%	3846 (28.3)	3386 (28.9)		648 (31.2)	3198 (27.7)	
100%	8112 (59.6)	7113 (60.6)		1289 (62.0)	6823 (59.2)	
Coverage change outcomes, among patients with partial Medicaid coverage^c^						
	Intervention clinic patients, partial coverage *N* = 5021	Control clinic patients, partial coverage *N* = 4319	Adjusted odds ratio^e^ (95% CI)	Patients with tool use, partial coverage N = 761	Patients without tool use, partial coverage *N* = 4260	Adjusted odds ratio^e^ (95% CI)
Gained coverage^d^, N (%)	3055 (60.8)	2426 (56.2)	**1.32 (1.14, 1.51)**	444 (58.3)	2611 (61.3)	**1.83 (1.64, 2.04)**
Lost coverage^d^, N (%)	2250 (44.8)	2173 (50.3)	**0.77 (0.68, 0.88)**	377 (49.5)	1873 (44.0)	**0.70 (0.53, 0.91)**

Data source = Medicaid enrollment data

**BOLD** = adjusted odds ratio significantly different from 1.0 (*p* < 0.05)

^a^χ^2^ tests of independence

^b^Period assessed = 12/1/2013–9/30/2015

^c^‘Partial Medicaid coverage’ = patient had Medicaid coverage for only part of the assessment period (12/1/2013–9/30/2015)

^d^‘Gained coverage’ = gained Medicaid after a period of being uninsured. ‘Lost coverage’ = lost Medicaid coverage after a period of insurance, including experiencing a gap in coverage

^e^Logistic GEE models clustered by clinic; adjusted for sex, age, race/ethnicity, primary language, federal poverty level, number of encounters in study period, new/established patient at first encounter, household member(s) on case (Y/N), length of time household established with clinic

**Table 3:** Follow-up visit and coverage status, patients with ≥1 uninsured visit within 6 months of intervention

	Intent To Treat (ITT)			Effect of Treatment on the Treated (ETOT) Intervention clinic patients only		
	Intervention clinic patients *N* = 1196	Control clinic patients *N* = 384	Adjusted odds ratio (95% CI)^a^	Patients with tool use *N* = 232	Patients without tool use *N* = 964	Adjusted odds ratio (95% CI)^a^
Returned for ≥1 visit in study period, N (%)	963 (80.5)	324 (84.4)	1.02 (0.77, 1.34)	202 (87.1)	761 (78.9)	1.78 (1.42, 2.23)
	Intervention patients, with a return visit *N* = 963	Control clinic patients, with a return visit *N* = 324	Adjusted odds ratio (95% CI)^a^	Patients with tool use, with a return visit *N* = 202	Patients without tool use, with a return visit *N* = 761	Adjusted odds ratio (95% CI)^a^
Insured by Medicaid at any return visit, N (%)	584 (60.6)	271 (83.6)	**0.42 (0.29, 0.61)**	130 (64.4)	454 (59.7)	2.00 (0.93, 4.31)
Uninsured at all return visits in study period, N (%)	356 (37.0)	51 (15.7)	**2.00 (1.37, 2.94)**	67 (33.2)	289 (38.0)	**0.49 (0.27, 0.90**)

Data source: EHR visit data

**BOLD** = adjusted odds ratio significantly different from 1.0 (p < 0.05)

Period assessed for uninsured visit = 12/1/2013–11/30/2014, Period assessed for return visits = patient-specific uninsured visit date through 11/30/2015

^a^Logistic GEE model clustered by clinic; adjusted for sex, age, race/ethnicity, primary language, federal poverty level, Medicaid identification number assigned (Y/N), household member(s) on case (Y/N), length of time household established with clinic, new patient at uninsured visit (Y/N)

We assessed *receipt of recommended healthcare services* among the subgroup of children recommended to receive each of the services based on age and sex (see Table 4 footnotes).

**Table 4:** CHIPRA measures of recommended care receipt in the 18 months after intervention

	Intent to Treat (ITT)					Effect of Treatment on the Treated (ETOT)(Intervention clinic patients only)				
	Intervention clinic patients N = 15024		Control clinic patients N = 12227			Patients with tool use N = 2240		Patients without tool use N = 12784		
	N eligible	N (%) received	N eligible	N (%) received	Adjusted OR (95% CI)	N eligible	N (%) received	N eligible	N (%) received	Adjusted OR (95% CI)
Well-child visits in first 15 months^a^	1141	483 (42.3)	1078	570 (52.9)**	**0.57 (0.38, 0.85)**	161	101 (62.7)	980	382 (39.0)**	**1.54 (1.06, 2.25)**
Well-child visit in 3rd-6th year^b^	4367	3926 (89.9)	3753	3389 (90.3)	1.09 (0.94, 1.26)	670	638 (95.2)	3697	3288 (88.9)**	1.44 (0.84, 2.46)
Adolescent well-care visit^c^	5899	5065 (85.9)	4429	3806 (85.9)	1.05 (0.71, 1.55)	917	824 (89.9)	4982	4241 (85.1)**	1.06 (0.82, 1.38)
BMI assessment for children/adolescents^d^	12101	11610 (95.9)	9581	9243 (96.5)*	0.81 (0.58, 1.14)	1927	1857 (96.4)	10,174	9753 (95.9)	1.07 (0.97, 1.18)
Human papillomavirus vaccine for female adolescents (HPV)^e^	927	358 (38.6)	689	262 (38.0)	1.10 (0.74, 1.64)	141	76 (53.9)	786	282 (35.9)**	**1.44 (1.12, 1.84)**
Chlamydia screening in women^f^	1596	450 (28.2)	1263	565 (44.7)**	**0.49 (0.36, 0.70)**	228	65 (28.5)	1368	385 (28.1)	1.24 (0.94, 1.62)
Childhood immunization status^g^										
Diphtheria, tetanus and pertussis (DTaP)^h^	1891	1458 (77.1)	1727	1362 (78.9)	0.92 (0.74, 1.15)	286	256 (89.5)	1605	1202 (74.9)**	**1.38 (1.09, 1.74)**
Inactivated polio (IPV)^i^	1891	1670 (88.3)	1727	1514 (87.7)	1.13 (0.87, 1.48)	286	277 (96.9)	1605	1393 (86.8)**	1.73 (0.83, 3.60)
Measles-Mumps-Rubella (MMR)^j^	1891	533 (28.2)	1727	656 (38.0)**	**0.61 (0.43, 0.86)**	286	92 (32.2)	1605	441 (27.5)	1.10 (0.69, 1.77)
Haemophilus influenza type b (Hib)^k^	1891	1681 (88.9)	1727	1517 (87.8)	1.23 (0.94, 1.60)	286	278 (97.2)	1605	1403 (87.4)**	1.83 (0.78, 4.26)
Hepatitis B^l^	1891	1697 (89.7)	1727	1544 (89.4)	1.15 (0.79, 1.69)	286	279 (97.6)	1605	1418 (88.4)**	2.03 (0.92, 4.44)
Varicella zoster virus (VZV)^m^	1891	1655 (87.5)	1727	1516 (87.8)	1.12 (0.76, 1.66)	286	269 (94.1)	1605	1386 (86.4)**	1.13 (0.74, 1.73)
Pneumococcal conjugate (PCV)^n^	1891	1424 (75.3)	1727	1258 (72.8)	1.20 (0.95, 1.52)	286	249 (87.1)	1605	1175 (73.2)**	1.20 (0.87, 1.65)
Hepatitis A^o^	1891	1640 (86.7)	1727	1515 (87.7)	1.04 (0.71, 1.53)	286	268 (93.7)	1605	1372 (85.5)**	1.10 (0.79, 1.53)
Rotavirus (RV)^p^	1891	1249 (66.1)	1727	1088 (63.0)	1.13 (0.89, 1.45)	286	233 (81.5)	1605	1016 (63.3)**	**1.45 (1.03, 2.04)**
Influenza^q^	1891	850 (44.9)	1727	807 (46.7)	0.91 (0.73, 1.13)	286	177 (61.9)	1605	673 (41.9)**	**1.47 (1.16, 1.86)**
Combination 10q^r^	1891	211 (11.2)	1727	243 (14.1)*	0.74 (0.38, 1.43)	286	51 (17.8)	1605	160 (10.0)**	1.22 (0.72, 2.05)
Immunizations for adolescents^s^										
Meningococcal^t^	1837	1400 (76.2)	1352	999 (73.9)	1.36 (1.00, 1.86)	304	255 (83.9)	1533	1145 (74.7)**	1.06 (0.78, 1.45)
Tetanus, diphtheria, pertussis/Tetanus, diphtheria (Tdap/Td)^u^	1837	1485 (80.8)	1352	1020 (75.4)**	**1.65 (1.26, 2.15)**	304	261 (85.9)	1533	1224 (79.8)*	0.93 (0.77, 1.12)
Combination 1^v^	1837	1355 (73.8)	1352	903 (66.8)**	**1.65 (1.23, 2.21)**	304	243 (79.9)	1533	1112 (72.5)*	0.93 (0.70, 1.23)

* χ^2^ Tests of independence *p* < .05

** χ^2^ Tests of independence *p* < .001

Odds ratios from logistic GEE model accounting for clustering within clinic, adjusted for sex (where appropriate), age (where appropriate), race/ethnicity, primary language, FPL, new vs. established patient status, family member on Medicaid case (Y/N), and length of time family had been established with the clinic

**BOLD** = adjusted odds ratio significantly different from 1.0 (*p* < .05)

^a^percentage of children aged 15 months with ≥6 well-child visits in first 15 months of life

^b^percentage of children ages 3 to 6 with ≥1 well-child visis

^c^percentage of children ages 12 to 21 with ≥1 comprehensive well-care visit

^d^percentage of children ages 3 to 17 with BMI documentation

^e^percentage of females aged 13 years with 3 doses of HPV vaccine by their 13th birthday

^f^percentage of sexually active females ages 16 to 20 with ≥1 test for chlamydia

^g^percentage of children aged 2 years with specific vaccines by their second birthday

^h^≥4 DTaP vaccinations with different dates of service, ≥42 days after birth

^i^≥3 IPV vaccinations with different dates of service, ≥42 days after birth

^j^≥1 MMR vaccine; or ≥ 1 measles & rubella vaccine plus 1 mumps vaccine; or ≥ 1 measles, ≥1 mumps, & ≥1 rubella vaccine

^k^≥3 Hib vaccinations with different dates of service, ≥42 days after birth

^l^≥3 HepB vaccinations with different dates of service

^m^≥1 VZV vaccination or history of varicella zoster

^n^≥4 PCV vaccinations with different dates of service, ≥42 days after birth

^o^≥1 HepA vaccination or history of HepA illness

^p^≥2 doses of 2-dose RV on different dates; or ≥ 3 doses of 3-dose RV on different dates; or ≥ 1 dose of 2-dose RV + ≥2 doses of 3-dose RV on different dates of service; ≥42 days after birth

^q^≥2 flu vaccinations with different dates of service, ≥180 days after birth

^r^children who were numerator compliant for all 10 above vaccinations

^s^percentage of adolescents aged 13 with specific vaccines by their 13th birthday

^t^≥1 meningococcal vaccination between 11th and 13th birthdays

^u^≥1 Tdap; or ≥ 1 Td; or ≥ 1 tetanus and ≥ 1 diphtheria between 10th and 13th birthdays

^v^adolescents who were numerator compliant for both above vaccinations

We used chi-square statistics to test between-group differences in the percentage of eligible children meeting each CHIPRA measure. We then estimated adjusted odds ratios for meeting each measure, comparing intervention versus control clinic patients (ITT) and patients in the intervention clinics with tool use versus those without tool use (ETOT). As before, odds ratios were estimated using adjusted logistic GEE models accounting for clinic clustering and adjusted for covariates (see footnotes, Table 4).

All quantitative analyses were performed in SAS software, v.9.4 (SAS Institute Inc., Cary, NC). Statistical significance was set at α < 0.05. The study was approved by the Oregon Health & Science University Institutional Review Board.

## Results

### Tool use

**Fig. 2:**
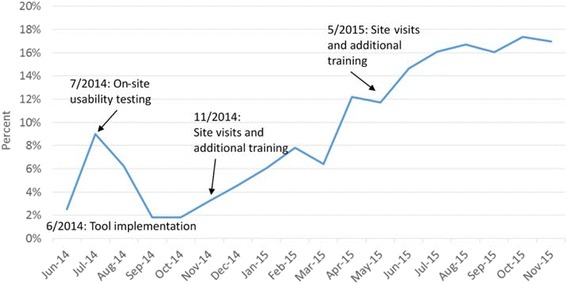
Tracking Form use by month with key study dates noted. Graphic representation of *Tracking Form* use over time throughout study period

The tracking tool was used for approximately 15% of pediatric patients with ≥1 clinical visit at an intervention site (Fig. 1 and Table 1). Tool use changed over time: an initial spike in use, which then followed by decreasing use over several months. After additional site visits and trainings, tracking tool use increased gradually but steadily throughout follow-up. By the end of follow-up, the tracking tool was being used on abou*t* 17% of pediatric patients per month (Fig. 2).

The characteristics of children at intervention clinics differed from those seen in matched control clinics. Intervention clinic patients were older, more commonly Hispanic and Spanish-speaking, and had lower household incomes (*p* < .001 for all). Within the intervention clinics, patients with tool use differed from those for whom the tool was not used, notably by ethnicity: 95% of patients with tool use were Hispanic, compared to 65% of within-clinic comparison patients; and 88% used Spanish as their primary language, compared to 51% (p < .001 for both, Table 1). Qualitative findings concurred that patients’ ethnicity and language drove tool use in the intervention clinics. Children for whom the tool was used were also older, had lower household incomes, were more likely to be established clinic patients, and had more clinical encounters during the study period compared to within-clinic comparison patients (p < .001).

Although designed for use with established pediatric patients, we observed tool use in two additional groups: (1) children with no clinical visits in the assessment period (*n* = 969), and (2) adult patients (*n* = 3207); see Additional file 2. We also qualitatively observed eligibility specialists using the tracking tool in unanticipated ways. For example, family members often came in together for insurance assistance, and eligibility specialists initiated entries into the tracking tool for everyone in the family. Additionally, almost one-third of adults with a Medicaid ID for whom the tracking tool was used did not share a case number with other household members, which indicated that the tracking tool was used for ‘single’ adults, not just family members of children being assisted.

### Barriers to tool use

Qualitative data suggest that tool use was affected by two factors. First, several initiatives were occurring concurrent to the study period, including Oregon’s 2014 Medicaid expansions; the US Health Resources and Services Administration (HRSA) funding for CHCs to extend insurance assistance to *all* community members; [34] and an alternative payment program requiring staff to document every type of patient interaction, including insurance assistance, using specified reporting methods.

These concurrent initiatives created a frenzy of insurance-oriented change, increased eligibility specialists’ workloads, and distracted staff from using the tracking tool consistently. In addition, our tools could not be easily adapted to the new workflow and reporting requirements involved in the HRSA and alternate payment model initiatives. Consequently, to avoid duplicate data entry, CHC staff often tracked insurance-related interactions in spreadsheets, rather than the tracking and roster tools. Eligibility specialists’ increased workloads also limited the time they had to use the roster tool to identify patients whose insurance was nearing expiration:

*It’s been really hard for me to get into that list. I know I should be working on it … even though I do the application online, it’s fairly time consuming ... So unless we block the schedule … there is not enough time to do the follow-up list.* [Clinic 4, Eligibility Specialist]

Second, as the state was flooded with expanded Medicaid insurance applications, some enrollees had their insurance ‘end dates’ electronically extended, so the end dates shown in our tools did not always coincide with the end dates an enrollee received from the state. This discrepancy confused eligibility specialists about when they should help patients reapply:

*We think that the insurance is supposed to be expired by January. Then we realize that it’s been extended three more months. When we check [in] three more months, the next day there’s another three more months. So my staff are like, “How can I do any follow up?” […] There is not a real and exactly accurate redetermination date for us to support our patients.* [Clinic 4, Eligibility Specialist Supervisor]

*We were excited because [the tools were] going to tell us when it’s time to renew. We’re going to be able to research all these patients and call them ... But then it didn’t really work. And then the front would schedule appointments because [the tool] would say it’s time to renew. But then when they would come [in] I would call and [the state] would say, no, it’s not time. So that was kind of a bummer. We thought it would work, and ... help not only the patient but, you know, us too.* [Clinic 5, Eligibility Specialist]

As a result, staff who were initially excited about using the tools could not trust the pop-up alerts to accurately notify them when patients needed to re-enroll, so they ignored the pop-ups and did not initiate tracking tool entries for additional patients.

### Tool impact on coverage and care utilization

In ITT analyses, intervention clinic patients had higher odds of gaining insurance coverage (adjusted odds ratio [aOR] = 1.32, 95% confidence interval [95%CI] 1.14–1.51) and lower odds of losing coverage (aOR = 0.77, 95%CI 0.68–0.88), compared to control clinic patients (Table 2). Similarly, in ETOT analyses, patients with tool use had higher odds of gaining insurance (aOR = 1.83, 95%CI 1.64–2.04), and lower odds of losing it (aOR = 0.70, 95%CI 0.53–0.91), versus patients in the clinic without tool use; Table 2.

In ITT analyses, uninsured intervention clinic patients had higher odds of being uninsured at all return visits (aOR = 2.00, 95%CI 1.37–2.94) and lower odds of being insured by Medicaid at any return visit (aOR = 0.42, 95% CI 0.29–0.61), compared to control clinic patients (Table 3). These relationships were reversed in ETOT analyses: uninsured patients with tool use had lower odds of being uninsured at all return visits (aOR = 0.49, 95%CI 0.27–0.90), and a trend toward higher odds of being insured by Medicaid (aOR = 2.00, 95% CI 0.93–4.31, *ns*), compared to patients without tool use (Table 3). The ETOT analysis also found higher odds of a return visit after an uninsured visit among treated patients (aOR = 1.78, 95%CI 1.42–2.23), Table 3.

### Tool impact on receipt of recommended care

In ITT analyses, intervention clinic patients had lower odds of receipt of several services versus control clinic patients (well-child visits in first 15 months, chlamydia screening, and MMR), but higher rates and odds of immunizations for adolescents (Table 4). In ETOT comparisons, tool use was associated with higher rates of receipt of most assessed services (Table 4). For example: 63% of eligible patients with tool use received recommended well-child visits by 15 months of age, versus 39% of within-clinic comparison patients (*p* < .001; aOR = 1.54, 95% CI 1.06–2.25); females for whom the tool was used were more likely to complete human papillomavirus (HPV) vaccination by age 13 (54%), versus 36% of within-clinic comparisons (p < .001; aOR = 1.44, 95%CI 1.12–1.84).

## Discussion

In this novel study, we partnered with stakeholders in a user-centered design process to build EHR-based insurance assistance tools for CHCs [21, 23, 24]. To our knowledge, this is the first study to build and test EHR-based tools designed to support primary care clinics as they assist patients with insurance enrollment.

In the 18 months post-implementation of our EHR-based tools, tool use rates were low. Our qualitative results suggest two important (and likely connected) factors that explain this outcome. Both were a result of this study occurring during historic healthcare reforms. First, federal initiatives incentivized the CHCs to use an insurance documentation system that was different from our tools, [34] making adoption of our tools less appealing. Second, swamped by the 44% Medicaid enrollment increase that occurred between July 2013 and March 2014, [35] Oregon electronically extended coverage expiration dates for many people already enrolled in public insurance without requiring that re-application paperwork be processed. This automatic extension of coverage compromised our ability to obtain accurate insurance coverage end dates for our tools and led to confusion and mistrust of the tools we developed. Such real-world factors are inherent to pragmatic trials and an important lesson in this nascent endeavor.

The most notable impact of the tools was in improving the odds that a child with a Medicaid ID either gained back coverage or did not lose coverage, in both ITT and ETOT analyses (Table 2). Among the uninsured children in this subsample, having a previous Medicaid ID indicated that a given child was likely eligible to regain coverage and may have just needed assistance from clinic staff using the tool. Among all uninsured children (those with or without prior Medicaid), it is likely that some children did not qualify for Medicaid for various reasons (e.g.*,* citizenship requirement). More children in the intervention group clinics may not have qualified, especially given the higher percentage of Hispanic patients and the lower percentage of children with Medicaid coverage in the intervention clinic group. This may explain why the ITT and ETOT analyses did not concur regarding return uninsured visits among uninsured children (ITT analyses showed higher odds of uninsured return visits; ETOT analyses showed lower odds of uninsured return visits). It is also important to note that the ETOT analyses were performed only using the intervention clinics. Restricting the sample to only intervention clinics, allows us to identify what impact the tool use had among those with access to those tools. Our ability to see a consistent population-level impact on receipt of recommended care in ITT analyses may have been hampered by low tool uptake [36]. However, it was promising to observe the consistency in the ETOT analyses with higher rates of every recommended healthcare service among patients with tool use versus those without tool use. This suggests that efforts to increase use of the tools could positively impact children’s receipt of recommended healthcare services in the future.

### Limitations

To accommodate stakeholder requests, our intervention was not randomly allocated to the four CHCs we initially recruited – all four received the intervention. Instead, we used propensity score techniques to identify four comparable matched CHCs. Although this approach is not as closely controlled as a more traditional randomized trial, it was chosen to suit this pragmatic, real-world evaluation. This approach was also more ethically appropriate for research in this setting because it enabled us to avoid recruiting CHCs who were very reluctant to be randomized into a control arm. Even using these matching methods, there were still significant differences between intervention and control sites, due to the small number of non-intervention CHCs available for matching. We attempted to address this imbalance through additional statistical regression adjustment. As with any observational study, there may have been unobserved differences between study groups, which in turn may have affected the study results. Due to EHRs not being structurally designed to link records for families, we could not assess whether tool use was impacted by the number of individuals assisted in a given family. The length of our follow-up period was 18 months, which may not have been sufficiently long to assess tool adoption and impact, including changes to CHC utilization and recommended care receipt. In addition, it was only technically feasible to quantify use of the tracking tool at the individual level, so we selected use of this tool to indicate that individual patients had been exposed to the intervention. While we evaluated use of the pop-up alert and roster tools in qualitative data collection, we were not able to assess whether use of these other features impacted the outcomes of interest. It is possible that non-tool use patients (our ETOT comparison group) had some action taken from the pop-up or roster tools that was not quantitatively tracked, thus underestimating our measures of tool effect.

While many of the ETOT results are promising, we caution against drawing long term conclusions based on these results in light of the more equivocal ITT findings. For EHR tools such as these to show convincing population-level impacts, they must be broadly adopted. Nonetheless, we did observe significant benefit among patients for whom clinic staff thought the tools would be useful.

### Next steps

Despite barriers to tool adoption, our results suggest several future potential benefits of EHR-based insurance assistance tools. CHC staff saw benefit in using the tools, especially for Hispanic, Spanish-speaking patients, and for some individuals beyond the study population (e.g., adults). It was encouraging to observe that the tools were used to assist many adults (*n* = 3207) as well as children (2240) in the intervention clinics. Many of these adults had a child who was also assisted, and qualitative data concurred that eligibility specialists often aided entire families. This finding led our team to obtain funding to develop and test similar tools for adults [37]. We also learned that designing and implementing EHR-based insurance assistance tools requires close collaboration with payers (e.g., Medicaid/CHIP), an infrastructure that can generate accurate data on insurance end dates, [38] and one that can be easily adapted to a changing insurance landscape.

## Conclusions

This study was the first to evaluate the feasibility and impact of developing EHR-based tools to help primary care clinics provide health insurance enrollment support for patients. Our results suggest that EHR-based tools have the potential to increase insurance enrollment, prevent coverage loss, and improve the receipt of recommended care. However, these results are only achieved when the tools are used; tool adoption overall was not high enough to fully assess population-level impacts. Nevertheless, encouraging findings from this novel study provide important lessons to improve such tools and increase their future adoption.

## Additional files

Additional file 1: IMPACCT electronic health record (EHR) tool descriptions. Illustrations of EHR study tools designed for use by community health center staff assisting patients with insurance enrollment. Data from fictitious patients were used when creating the illustrations. (DOCX 10958 kb)

Additional file 2: Demographic and encounter characteristics, individuals with tracking tool use outside of the study population. Table presenting sex, age, race-ethnicity, primary language, and percent of FPL characteristics of two groups outside the study population for whom the *Insurance Tracking Form* was used (children with no clinical visits in the assessment period, *n* = 969; and, adult patients, *n* = 3207). (DOCX 16 kb)

## Abbreviations

aOR: adjusted odds ratio
CHC: community health center
CHIP: Children’s Health Insurance Program
CHIPRA: Children’s Health Insurance Program Reauthorization Act
CI: confidence interval
EHR: electronic health record
GEE: generalized estimating equation
HPV: human papillomavirus
HRSA: Health Resources and Services Administration
ID: identification number
ITT: intent-to-treat
US: United States
