# Supplementary material for: Electronic health record tools to assist with children’s insurance coverage: a mixed methods study
Source: BMC Health Serv Res. 2018 May 10;18:354. doi: 10.1186/s12913-018-3159-x (PMC5946500; doi:10.1186/s12913-018-3159-x)
Supplement: Supplementary file 1 — IMPACCT electronic health record (EHR) tool descriptions. Illustrations of EHR study tools designed for use by community health center staff assisting patients with insurance enrollment. Data from fictitious patients were used when creating the illustrations. (DOCX 10958 kb) [file 12913_2018_3159_MOESM1_ESM.docx]

**Additional File 1. The IMPACCT Electronic Health Record (EHR) Tools.**

The tools were designed for use by CHC staff assisting patients with insurance enrollment. Data from fictitious patients were used when creating the following illustrations.

**Tracking & Documentation Form for Insurance Applications** *(Tracking Form*; Figure 1*)* was designed to record relevant insurance application data, including fields to capture status of the application (submitted, pending, approved, and denied), the date application was submitted, the insurance type, the enrollment assister who opened the form, and a free text field for entering notes about missing information and/or action steps for the next appointment. In addition, the form also included a field for entering a follow-up date to support the workflows for checking the status of an application.

**Figure 1.** Tracking and Documentation Form for Insurance Applications


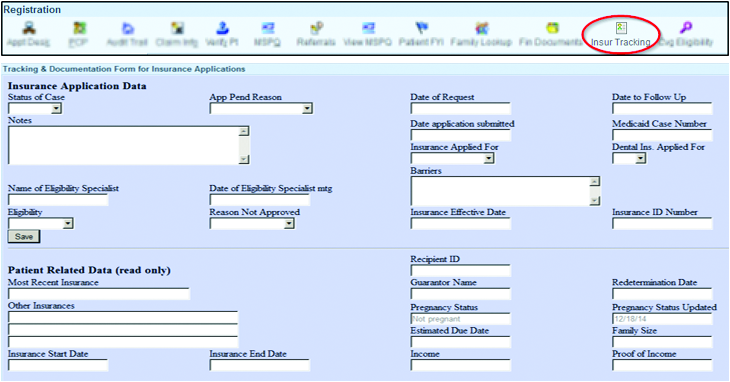


© 2016 Epic Systems Corporation. Used with permission.

**Reporting / Roster Tools** (*Roster Tool*; Figures 2 through 4) which enabled clinic staff to create a daily report, listing patients who have a *Tracking Form* and 1) for whom insurance assistance has already been initiated and are in need of follow-up (IMPACCT Follow-up Report), or 2) who have a clinical appointment coming up and might need insurance enrollment support (IMPACCT Upcoming Appointments Report). A *Tracking Form* for each patient included on the report was easily accessible from the reports, facilitating the updating of the form.

**Figure 2.** Reporting / Roster Tools – IMPACCT Follow-up Report

**
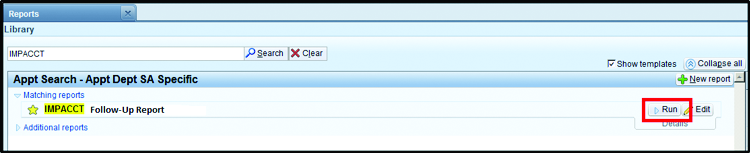
**

© 2016 Epic Systems Corporation. Used with permission.

**Figure 3.** Reporting / Roster Tools – IMPACCT Upcoming Appointments Report


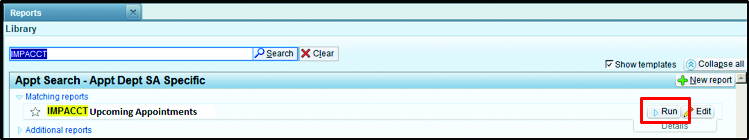


© 2016 Epic Systems Corporation. Used with permission.

**Figure 4.** IMPACCT Follow-up Roster


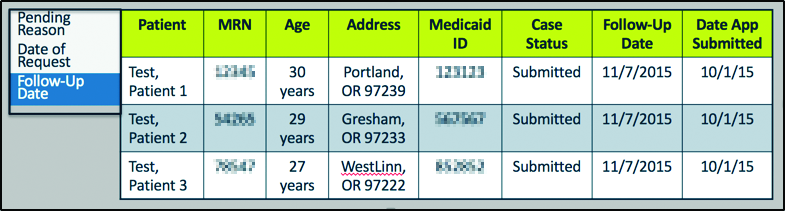


© 2016 Epic Systems Corporation. Used with permission. (Fictitious patient data).

**Insurance Coverage Information (pop-up warnings, coverage verification option, appointment information insurance data, Department Appointments Report):** To meet clinics’ need to identify patients in need of health insurance application assistance, we made health insurance information visible in a few different locations in the EHR Registration module:

- **Pop-up Alerts (Figure 5):** Notifications that popped up when clinic staff closed the Registration page of, scheduled an appointment for, or checked in a patient whose health insurance was soon to expire or when a patient appeared eligible but not enrolled in public health insurance.

**Figure 5.** Pop-up Alert


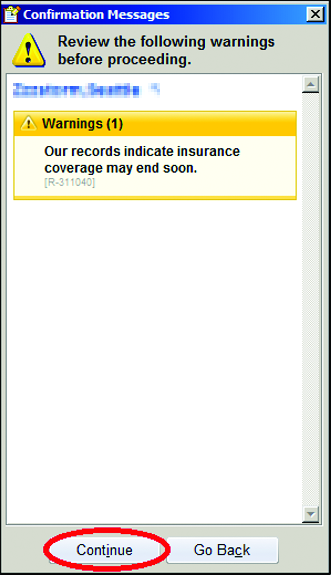


© 2016 Epic Systems Corporation. Used with permission. (Fictitious patient data).

- **Coverage verification option (Figure 6):** A new dropdown menu option under *Coverage Verification* that allows staff to indicate that public health insurance coverage may soon be expiring for a patient.

**Figure 6.** Coverage Verification Option


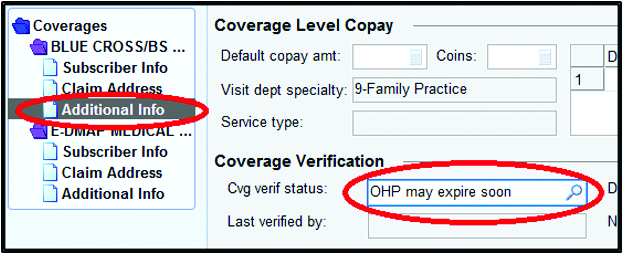


© 2016 Epic Systems Corporation. Used with permission.

- **Appointment Information Insurance Data (Figure 7):** New fields in the Appointment Information section that display health insurance data (Medicaid Redetermination Date, Medicaid Application Status, Insurance Assistant Appointment).

**Figure 7.** Appointment Information Insurance Data

**
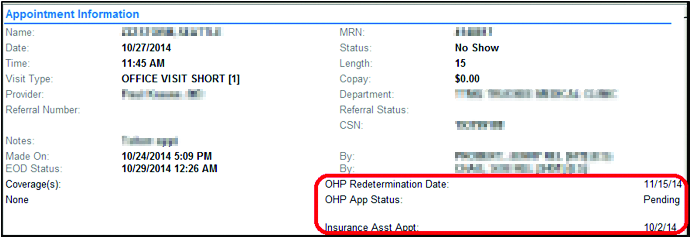
**

© 2016 Epic Systems Corporation. Used with permission. (Fictitious patient data).

**IMPACCT DAR (Figures 8 and 9):** A special Department Appointments Report (DAR) created to highlight insurance expiration data (Medicaid Redetermination Date, Medicaid Application Status, Insurance Specialist Appointment Date). Users can add any of these new data elements to an existing, customized DAR.

**Figure 8.** Accessing IMPACCT Department Appointment Report


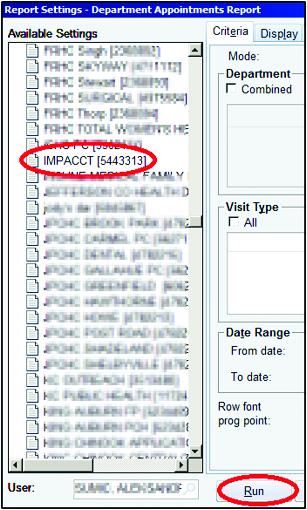


© 2016 Epic Systems Corporation. Used with permission.

**Figure 9.** IMPACCT Department Appointment Report


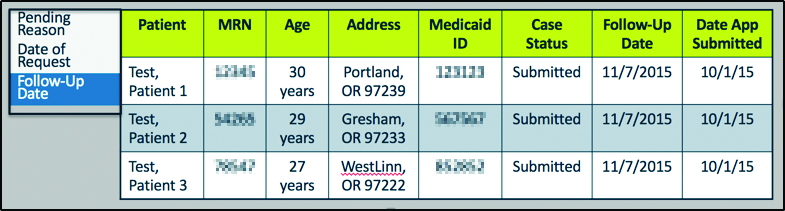
© 2016 Epic Systems Corporation. Used with permission. (Fictitious patient data).
