# Supplementary material for: Electronic health record tools to assist with children’s insurance coverage: a mixed methods study
Source: BMC Health Serv Res. 2018 May 10;18:354. doi: 10.1186/s12913-018-3159-x (PMC5946500; doi:10.1186/s12913-018-3159-x)
Supplement: Supplementary file 2 — Demographic and encounter characteristics, individuals with tracking tool use outside of the study population. Table presenting sex, age, race-ethnicity, primary language, and percent of FPL characteristics of two groups outside the study population for whom the Insurance Tracking Form was used (children with no clinical visits in the assessment period, n = 969; and, adult patients, n = 3207). (DOCX 16 kb) [file 12913_2018_3159_MOESM2_ESM.docx]

**Additional File 2**

| **Demographic and encounter characteristics, individuals with *Insurance Tracking Form* use outside of the study population** | | |
| --- | --- | --- |
|  | **Children with no clinical visit**  **N=969** | **Adults, with or without clinical visits**  **N=3,207** |
| **Gender** |  |  |
| Male | 487 (50.3) | 1133 (35.3) |
| Female | 482 (49.7) | 2074 (64.7) |
| **Age** (at first tool usage) |  | *--* |
| <1 yr | 6 (0.6) | -- |
| 1-4 | 89 (9.2) | -- |
| 5-12 | 446 (46.0) | -- |
| 13-17 | 316 (32.6) | -- |
| 18-19 | 112 (11.6) | -- |
| 20-34 | -- | 1034 (32.2) |
| 35-49 | -- | 1447 (45.1) |
| 50-64 | -- | 682 (21.3) |
| 65 and older | -- | 44 (1.4) |
| **Race-ethnicity** |  |  |
| Hispanic | 897 (92.6) | 2770 (86.4) |
| Non-Hispanic white | 52 (5.4) | 283 (8.8) |
| Non-Hispanic other | 5 (0.5) | 133 (4.2) |
| Unknown | 15 (1.6) | 21 (0.6) |
| **Primary language** |  |  |
| Spanish | 812 (83.8) | 2569 (80.1) |
| English | 133 (13.7) | 584 (18.2) |
| Other | 4 (0.4) | 36 (1.1) |
| Unknown | 20 (2.1) | 18 (0.6) |
| **Percent of FPL** |  |  |
| ≤138 | 862 (89.0) | 2827 (88.2) |
| 139-199 | 36 (3.7) | 243 (7.6) |
| ≥200 | 15 (1.6) | 65 (2.0) |
| Unknown | 56 (5.8) | 72 (2.3) |
| Note: Pediatric patients defined as ages 0-19 at first study encounter or tool usage date. Adult patients were age ≥20 at first study encounter or tool usage date. | | |
